# C-mii: a tool for plant miRNA and target identification

**DOI:** 10.1186/1471-2164-13-S7-S16

**Published:** 2012-12-07

**Authors:** Somrak Numnark, Wuttichai Mhuantong, Supawadee Ingsriswang, Duangdao Wichadakul

**Affiliations:** 1 Information Systems Laboratory, National Center for Genetic Engineering and Biotechnology (BIOTEC), 113 Thailand Science Park, Phaholyothin Road, Klong 1, Klong Luang, Pathumthani, Thailand; 2 Enzyme Technology Laboratory, National Center for Genetic Engineering and Biotechnology (BIOTEC), 113 Thailand Science Park, Phaholyothin Road, Klong 1, Klong Luang, Pathumthani, Thailand

## Abstract

**Background:**

MicroRNAs (miRNAs) have been known to play an important role in several biological processes in both animals and plants. Although several tools for miRNA and target identification are available, the number of tools tailored towards plants is limited, and those that are available have specific functionality, lack graphical user interfaces, and restrict the number of input sequences. Large-scale computational identifications of miRNAs and/or targets of several plants have been also reported. Their methods, however, are only described as flow diagrams, which require programming skills and the understanding of input and output of the connected programs to reproduce.

**Results:**

To overcome these limitations and programming complexities, we proposed C-mii as a ready-made software package for both plant miRNA and target identification. C-mii was designed and implemented based on established computational steps and criteria derived from previous literature with the following distinguishing features. First, software is easy to install with all-in-one programs and packaged databases. Second, it comes with graphical user interfaces (GUIs) for ease of use. Users can identify plant miRNAs and targets via step-by-step execution, explore the detailed results from each step, filter the results according to proposed constraints in plant miRNA and target biogenesis, and export sequences and structures of interest. Third, it supplies bird's eye views of the identification results with infographics and grouping information. Fourth, in terms of functionality, it extends the standard computational steps of miRNA target identification with miRNA-target folding and GO annotation. Fifth, it provides helper functions for the update of pre-installed databases and automatic recovery. Finally, it supports multi-project and multi-thread management.

**Conclusions:**

C-mii constitutes the first complete software package with graphical user interfaces enabling computational identification of both plant miRNA genes and miRNA targets. With the provided functionalities, it can help accelerate the study of plant miRNAs and targets, especially for small and medium plant molecular labs without bioinformaticians. C-mii is freely available at http://www.biotec.or.th/isl/c-mii for both Windows and Ubuntu Linux platforms.

## Background

MicroRNAs (miRNAs) are a class of small, non-coding, single-stranded RNA molecules of 18-22 nucleotides. In various species, they play roles in gene regulation by targeting mRNAs at the post-transcriptional level [1]. In plants, miRNAs are involved in organ development and environmental responses [2-4]. Although several miRNA and target prediction tools are available [5,6], the number of tools customized for plant miRNA and target analysis is limited. Among them are microHARVESTER, a web server for identifying plant miRNAs [7]; the miRU [8], psRNATarget [9], and TAPIR [10] web servers for identifying plant miRNA targets; a web-based toolkit for the analysis of plant small RNAs [11]; and the miRTour web server for plant miRNA and target prediction [12]. Most of these public web servers limit the number of input sequences and focus on only miRNA or target identification. Target-align [13] was recently proposed for plant miRNA target identification and developed as both web and command line versions. Even though several studies in computational identification of plant miRNAs and their targets are available [14-25], their methods were mainly presented as flow diagrams of connected programs (e.g., BLAST [26], UNAFold [27]). To follow the same steps, users need to install these programs, understand their usage, comprehend the meaning and format of the results, and have the programming experience for connecting them together.

Taking into account all these computational steps and criteria for plant miRNA and target identification [28-38], we developed C-mii, a standalone software package with graphical user interfaces for identifying, manipulating, and analyzing plant miRNAs and targets. C-mii is implemented as an all-in-one Java package weaving together sequence similarity search, secondary structure folding, automatic stem-loop identification and manipulation, and functional and gene ontology (GO) annotation. In addition, it comes pre-installed with databases of proteins, non-coding RNAs, and mature miRNAs. C-mii expects a set of nucleotide sequences (e.g., cDNAs, Expressed Sequence Tags (ESTs), Genome Survey Sequences (GSS)) in FASTA format as input. The identification steps are divided into miRNA and target identification pipelines. Users can customize parameter settings for each step of the identification, and filter and manipulate the results according to various biological criteria.

## Materials and methods

### Workflow overview

**Figure 1:**
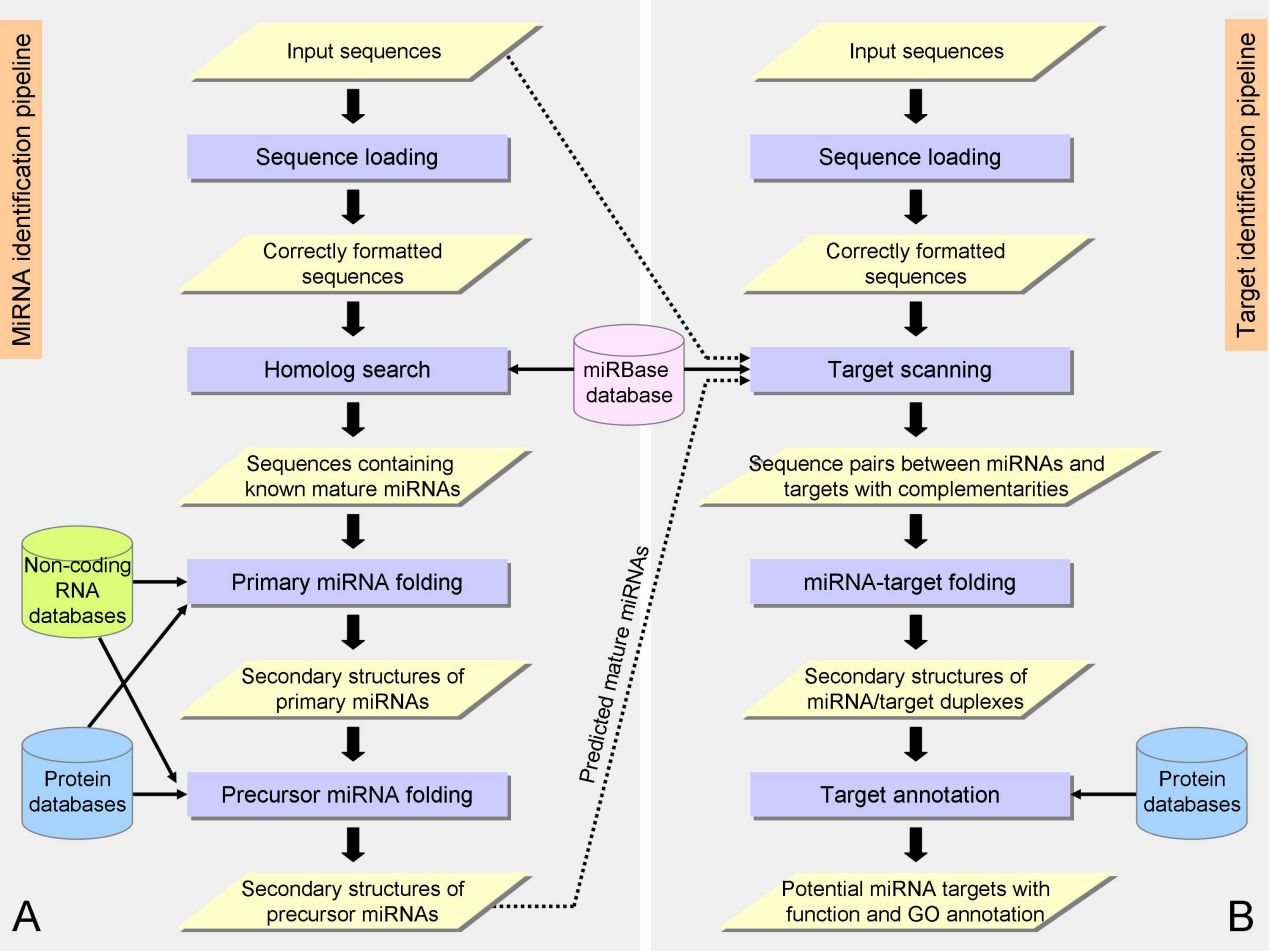
**Workflow of C-mii**. (A) miRNA identification pipeline, and (B) target identification pipeline. The dashed lines represent the optional usage of input sequences and predicted mature miRNA sequences from the miRNA identification by the target scanning.

Figure 1 shows the overall computational steps of C-mii with two pipelines consisting of miRNA and target identifications. The miRNA identification pipeline includes the consecutive execution of four main modules: sequence loading and validation, homolog search, primary miRNA folding, and precursor miRNA folding. The sequence validation checks the uploaded file format and excludes sequences longer than 3000 nt or with non-nucleotide characters. The homolog search determines whether input sequences contain homologous mature miRNAs. This module is implemented based on BLAST and sequence scan with user-selected mature miRNAs pre-installed from miRBase [39,40]. The primary miRNA folding module predicts the secondary structure folding of input sequences containing homologous miRNAs. Precursor miRNA folding, the last module of the pipeline, extracts and re-predicts the stem-loop structures from the primary miRNA structures. Then, for validation, it examines whether these structures satisfy the constraints of plant precursor miRNA biogenesis. Both the primary and precursor miRNA folding modules employ UNAFold to predict secondary structure folding. The Rfam [41] and UniProt [42] databases are also incorporated, with each step allowing users to remove sequences that are other types of RNAs and protein-coding sequences, respectively.

The target identification pipeline consists of four consecutive modules: sequence loading and validation, target scanning, miRNA-target folding, and target annotation. The sequence loading module is the same as that for miRNA identification, but accepts input sequences of longer lengths (≤ 20,000 nt). The target scanning module determines if input sequences contain the complementary sites to mature miRNAs of interest. Implementation of this module is similar to the homolog search except for the use of reverse-complement mature miRNAs as queries for BLAST. The miRNA-target folding module utilizes UNAFold to predict the secondary structure and free energy of miRNA:target duplexes. This module was introduced to C-mii to help refine the target identification result. The last module, target annotation, supplies function and gene ontology (GO) for the potential target sequences. Along the computational steps of both pipelines, users can customize parameters for their execution, select databases of interest, and explore and filter the results.

### Pre-installed databases

The pre-installed databases of C-mii are divided into three categories. First, the mature miRNA database, miRBase release 16, was incorporated for homolog search. Second, the non-coding RNA database, Rfam 10 with removed miRNAs, was introduced for removing other types of RNAs. Third, the protein databases, the UniProtKB/Swiss-Prot release 2010_12 and UniProtKB/TrEMBL release 2011_01, were incorporated for removing protein-coding sequences in the primary and precursor miRNA folding steps and for identifying gene functions during target annotation. These databases were pre-processed using the formatdb program in the BLAST package. Users can update these databases from our web site via a menu in C-mii. Furthermore, users can integrate their own protein and non-coding RNA databases into the system by following the pre-processing steps documented on the C-mii web site.

### Pre-installed software packages

To ease its installation and usage, C-mii was designed as a complete package containing all required software, including BLAST, Java Development Kit (JDK), Perl, Python, UNAFold, and Ghostscript [43], which can be customized during installation. To explore conservation and co-evolution among the predicted and known precursors or mature miRNAs of an miRNA family, CLUSTAL W [44], MUSCLE [45] and Jalview [46] were pre-installed for performing and visualizing the multiple sequence alignment of selected identification results. In addition, we have deployed prefuse visualization toolkit [47], ICEpdf Viewer [48], and JFreeChart [49] for visualizing GO trees, the secondary structure folding of primary and precursor miRNAs, and the infographics, respectively.

## Results and discussion

C-mii is composed of two pipelines for plant miRNA and target identifications, which could be used autonomously or consecutively. The functionalities of these pipelines have been described with biological rationales and a running example (visit http://www.biotec.or.th/isl/c-mii/documentation.php under "C-mii running example section" to see screenshots of all steps).

### MiRNA identification pipeline

Taking into account the computational steps and criteria for plant miRNA identification as described previously [28,29,31], the miRNA prediction menu consists of four consecutive submenus starting from sequence loading, homolog search, primary miRNA folding, and precursor miRNA folding. Users need to build a new project before uploading nucleotide sequences in a FASTA file. Sequences longer than 3,000 nt or containing characters other than A, T, C, G, U, and N will be excluded. As a running example, we built a TAIR10 cDNA project of 33,602 sequences. Due to sequence validation, 30,707 sequences remained as input for the homolog search.

#### Homolog search

**Figure 2:**
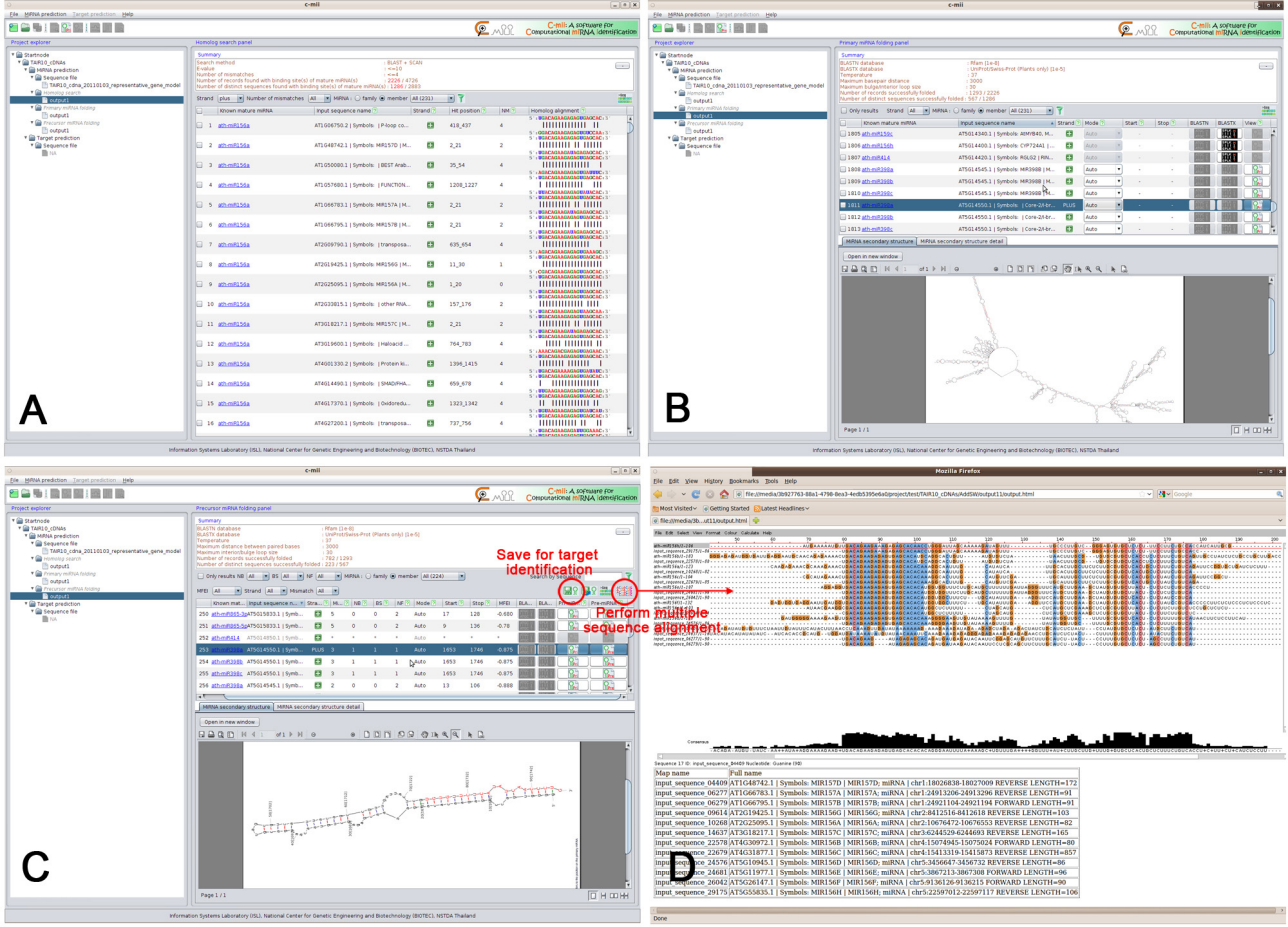
**MiRNA identification results**. (A) homolog search results, (B) primary miRNA folding results, (C) precursor miRNA folding results with various filters corresponding to suggested criteria in previous literatures, (D) multiple sequence alignment of known and predicted precursor miRNAs of miR156 family.

The homolog search module helps users identify input sequences that contain mature miRNA sequences from miRBase. In this step, users can select mature miRNAs of multiple plants from miRBase to be used as source mature miRNAs for the identification process. The identification methods include sequence scans with and without BLAST. The sequence scan with BLAST is much faster, but with the trade-off of possibly missing matches due to the word size limitation of BLASTN, which needs to be 4 or greater. Users can also customize the E-value (≤ 10 by default) of BLASTN, the number of allowed mismatches (≤ 4 by default) between a source mature miRNA and its homolog in an input sequence, and the number of processors automatically detected by C-mii for running the homolog search. Figure 2A shows the homolog search results for *Arabidopsis *mature miRNAs from miRBase with the 30,707 TAIR10 cDNA sequences with default parameter settings. Using the plus strand only, 1286 *Arabidopsis *cDNAs of 129 miRNA families and 231 members were identified. We selected all these sequences as input for the primary miRNA folding module.

#### Primary miRNA folding

An miRNA gene needs to be non-coding and have a stem-loop precursor in its secondary structure [31]. The primary miRNA folding module helps users remove protein-coding sequences and other types of non-coding RNAs from input sequences, and predicts the secondary structure folding of primary miRNAs (pri-miRNAs) from the remaining sequences. The removal of unwanted sequence types is based on BLASTX and BLASTN against protein and non-coding RNA databases, selectable by users. Users can also adjust the E-value to limit the number of search results. The lower the E-value, the larger the number of sequences remains for secondary structure folding. C-mii sets the default E-value of BLASTN against Rfam as 1E-8 according to our previous benchmark [50]. Users may also customize the folding temperature, maximum base pair distance, and maximum bulge or interior loop size of UNAFold.

Figure 2B shows the primary miRNA folding results with default parameter settings with the exception of the BLASTX E-value ≤ 1E-5. Sequences with a clickable BLASTN or BLASTX box are sequences that hit with other sequence types. By clicking on these boxes, users can explore their E-values and hit sequences. The "Only results" check box allows users to filter out these sequences. For the remaining sequences, users can interactively explore their secondary structures and minimal free energies (MFEs) predicted by UNAFold, minimal folding free energy indices (MFEI) [30], sequence lengths, and nucleotide and GC content. The "Mode" column provides two options for extracting stem-loop precursors from a secondary structure of a primary miRNA. C-mii decides the cleavage positions for users in *Auto *mode. The *Manual *mode allows users to specify the start and stop cleavage positions. From the results of our running example using the plus strand only, 567 *Arabidopsis *cDNAs in 124 miRNA families and 224 members remained. All these sequences were selected with *Auto *mode for the precursor miRNA folding module.

#### Precursor miRNA folding

The precursor miRNA folding module helps users (1) extract the stem-loop structures from the secondary structures of pri-miRNAs, (2) remove stem-loop sequences that hit protein-coding sequences and other types of non-coding RNAs, (3) re-predict the secondary structure folding of the extracted stem-loop sequences, and (4) verify the predicted structures with previously suggested criteria. With the *Auto *mode setting from the previous step, the precursor miRNA folding module will cleave a pri-miRNA structure from the start position of the found homologous miRNA to the end position of its duplex miRNA* with two-nucleotide 3' overhangs. The extracted stem-loop sequences are screened against the protein and non-coding RNA databases again. UNAFold is then reapplied to the remaining sequences to predict the secondary structure folding of precursor miRNAs (pre-miRNAs). Users can customize the same set of parameters as in the primary miRNA folding step.

Figure 2C shows the precursor miRNA folding results with default parameter settings with the exception of the BLASTX E-value ≤ 1E-5. Based on previously reported criteria [28,31], structures with multi-loops or mature miRNA sequences not located within one arm will be automatically removed by C-mii. Besides MFEIs, users can filter the results by restricting the number of two-nucleotide 3' overhangs, the number of mismatches between miRNA:miRNA* duplexes, the number of bulges, and bulge sizes as proposed in [31]. Users can browse through the predicted secondary structures, select potential miRNAs based on filters, save them for target identification, and export them as an archive. Users may also perform multiple sequence alignments among the identified and known precursor or mature miRNAs of the same family to explore their conservation and evolution (Figure 2D). Using the plus strand only, 223 *Arabidopsis *cDNAs in 103 miRNA families and 197 members were finalized as potential miRNAs from 30,707 TAIR10 cDNAs (see System benchmarking and validation section for the detailed discussion).

### Target identification pipeline

C-mii's target prediction menu consists of four submenus: sequence loading, target scanning, miRNA-target folding, and target annotation. Users may build a new project for target identification only or continue the project from the miRNA identification pipeline. Users may also upload a new set of nucleotide sequences in FASTA format or reuse the uploaded sequences from the miRNA identification process. However, the acceptable length of an input sequence for target identification is extended to 20,000 nt. Besides sequence uploading, users also need to select mature miRNAs of interest from miRBase and/or from predicted mature miRNAs saved from the miRNA identification pipeline. In our running example, 434 sequences previously reported as miRNA-specific targets in *Arabidopsis *were uploaded to a new project and all mature *Arabidopsis *miRNAs from miRBase were selected for target scanning.

#### Target scanning

**Figure 3:**
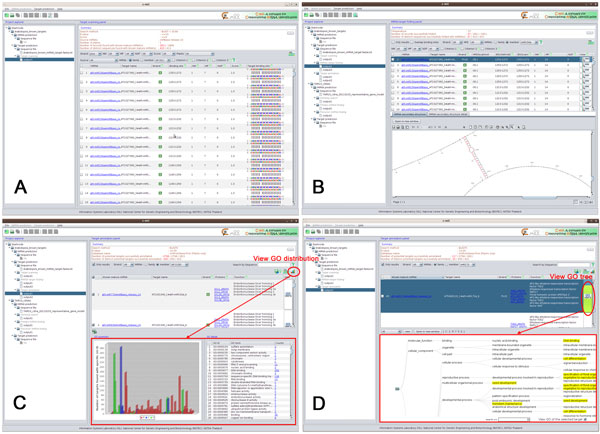
**Target identification results**. (A) target scanning results, (B) miRNA-target folding results, (C) overall target annotation results with GO summary, and (D) individual target annotation result with GO tree.

Based on plant-miRNA target binding through perfect or nearly-perfect complementarities [32,34], the target scanning module allows users to scan for complementary sites of selected mature miRNAs on input sequences. Based on criteria used in Rhoades et al. [33,35], users can customize the binding score as described in [38]. The scanning methods are the same as that of the homolog search with the reverse-complement of mature miRNAs used as queries for BLAST. Users can filter the results by the number of GU pairs, binding score, and mismatched position of interest. In addition, as the specific positions of mismatches affect miRNA targeting [22,36,37], C-mii also allows users to filter for target sequences whose miRNA binding site has no more than one mismatch at positions 1-9, no more than two consecutive mismatches, and no mismatches at positions 10 and 11. Figure 3A shows the target scanning results of our running example with default parameter settings. Using the plus strand only, 410 out of 434 sequences were identified as miRNA binding sites for 61 miRNA families and 133 members. All of them were selected as input for miRNA-target folding.

#### MiRNA-target folding

The miRNA-target folding module helps users refine their scanning results. It uses UNAFold to predict the secondary structures of miRNA:target duplexes, MFEs for hybridization, and binding positions of miRNA-target pairs. Users can specify the temperature that might affect miRNA:target duplex formation. Figure 3B shows the miRNA-target folding results of our running example using the default 37 ºC. From the results, users can determine potential miRNA targets based on the binding score from target scanning, the number of mismatches and G:U pairs between miRNA and its potential target from miRNA-target folding, mismatched positions, MFEs, and overlapped binding positions between the two steps. In our example, with the plus strand only, all 410 sequences remained for target annotation.

#### Target annotation

The target annotation module supplies function and gene ontology (GO) for potential targets selected from the previous step. Users can choose a protein database and customize the E-value and number of hits for BLASTX. Figure 3C shows the target annotation results of 400 out of 410 sequences using default parameter settings (see System benchmarking and validation section for the detailed discussion). From the results, users may explore GO annotation for a set of targets. By clicking on "Graph icon," C-mii calculates and visualizes the distribution of selected targets' GO IDs, colored by GO molecular function (F), biological process (P), and cellular component (C). From this view, users may also explore potential targets annotated with the same GO. The "Go View" allows users to investigate GO annotation of an individual potential target via a GO tree (Figure 3D). Users may also follow web links to public databases of known miRNAs and target functions.

### Summary views

**Figure 4:**
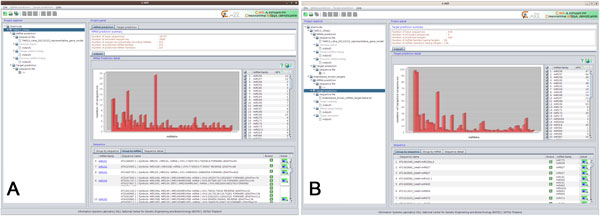
**Summary view**. (A) miRNA prediction view, (B) target prediction view.

The project summary view provides users with the overall number of identified miRNA families and targets, which can be exported as a report and linked back to the detailed identification results. The miRNA prediction view (Figure 4A) shows the overall number of input sequences, excluded sequences, sequences potentially encoding miRNAs, and the identified miRNA families and members. The infographics highlight the distribution of the identified miRNA families. The "Group by sequence" and "Group by miRNA" options allow users to explore the identified miRNAs for the same sequence and the list of sequences identified for the same miRNA. The "Detail" icon allows users to follow the link back to the results of precursor miRNA folding. Similarly, the target prediction view (Figure 4B) shows the overall number of input sequences, excluded sequences, the identified targets, and source miRNA families and members having targets.

### System validation and benchmarking

For system benchmarking, we applied C-mii to the four datasets: (i) TAIR10 cDNAs (33,602 sequences), (ii) TAIR10 miRNAs (176 sequences), (iii) *Arabidopsis *precursor miRNAs from miRBase 16 (213 sequences), and (iv) plant RNAs that are not miRNAs from Rfam 10 (16,219 sequences) (see Additional files 1, 2, 3, 4 for these sequences). The source of mature miRNAs was *Arabidopsis *miRNAs from miRBase 16. With default parameter settings except the E-value ≤ 1E-5 for BLASTX against the UniProt/Swiss-Prot protein database, Table 1 shows the number of remaining input sequences filtered for plus strand from each step of miRNA identification on the four datasets.

**Table 1:** Number of remaining input sequences from each step of miRNA identification on the four datasets

	TAIR10 (cDNAs)	TAIR10 (miRNAs)	miRBase 16 (*Arabidopsis *only)	Rfam 10 (all plant RNAs except miRNAs)
**miRNA identification steps**	33,602	176	213	16,219
1. Sequence loading	30,707	176	213	15,822
2. Homolog search	1286	175	213	31
3. Primary miRNA folding	567	173	209	0
4. Precursor miRNA folding	223	164	195	0

Table 2 shows the number of true and false miRNAs identified by C-mii for the above datasets except for the TAIR10 miRNAs dataset, which is the subset of TAIR10 cDNAs. From the TAIR10 cDNA dataset, 164 out of 223 cDNAs selected by the precursor miRNA folding step were true positives (TP), consistently annotated as miRNAs in TAIR10. The remaining 59 cDNAs were considered false positives (FP). Twelve TAIR10 miRNAs were missing from the identification results and were considered as false negatives (FN) (see Additional file 5 for the list of these TP, FP, and FN of TAIR10 cDNAs). True negatives (TN) were the non-miRNA input sequences that were excluded from the miRNA identification results. With *Arabidopsis *precursor miRNAs from miRBase, 195 out of 213 miRNAs were correctly identified while the remaining 18 miRNAs were missing and considered as FN (see Additional file 6 for list of these FN). All plant RNAs that are not miRNAs from Rfam 10 were excluded from the identification results and considered as TN. The positive predictive value (PPV) for the TAIR10 cDNA dataset = 164/(164+59) = 0.7354 while the negative predictive value (NPV) = 30,472/(12 + 30,472) = 0.9996. The sensitivity of the TAIR10 cDNA dataset = 164/(164+12) = 0.9318 whereas the specificity = 30,472/(59 + 30,472) = 0.9981. The PPV, NPV, sensitivity, and specificity of the combined datasets were 0.8589, 0.9994, 0.9229, and 0.9987, respectively.

**Table 2:** Number of TP, FP, FN, and TN of miRNA identification on the three datasets

Data sets	Number of sequences	Number of identified miRNAs	TP	FP	FN	TN
TAIR10 cDNAs	30,707	223	164	59	12	30,472
miRBase 16.0 (*Arabidopsis *only)	213	195	195	0	18	0
Rfam 10 (all plant RNAs except miRNAs)	15,822	0	0	0	0	15,822
Total	46,742	418	359	59	30	46,294

The previously reported 434 sequences of miRNA-associated targets of *Arabidopsis *(from 183 distinct TAIR10 gene loci and 49 *Arabidopsis *miRNA families) were used for benchmarking the target identification (see Additional file 7 for these sequences). The sensitivity was measured with two sets of parameter settings. The default settings included a binding score ≤ 4 in *Target scanning*, a folding temperature = 37 ºC for UNAFold in *MiRNA-target folding*, and an E-value ≤ 1E-20 for BLASTX against the plant-only UniProtKB/Swiss-Prot database in *Target annotation*. The customized settings used a binding score ≤ 6 in *Target scanning *and an E-value ≤ 1E-5 instead of 1E-20 in *Target annotation*. With mature *Arabidopsis *miRNAs from miRBase, C-mii identified 400 out of 434 miRNA-associated target sequences using default settings and the plus strand filter. Twenty-four sequences were lost due to the overly limited binding score, which was ≤ 4 in the default settings. The other ten miRNA-associated targets of seven distinct TAIR10 gene loci were lost in the annotation step; four out of seven were trans-acting siRNAs while the other three had too large hit E-values (> 0.1). Table 3 shows the number of known target sequences remaining from each step of target identification. With the use of the UniProtKB/Swiss-Prot protein database, the sensitivity of the identification calculated as TP/(TP + FN) was 0.922 and 0.933 for the default and customized settings respectively.

**Table 3:** Number of remaining input sequences from each step of target identification on the previously reported miRNA-associated target sequences of *Arabidopsis*

Step/Number of remaining sequences		Default settings	Customized settings
Number of input sequences		434	434

1. Sequence loading		434	434

2. Target scanning*		410	430

3. miRNA-target folding		410	430

4. Target annotation**	UniProtKB/Swiss-Prot	400	405

	UniProtKB/TrEMBL	406	427

* The default and customized binding scores for target scanning were ≤ 4 and ≤ 6, respectively.

** The default and customized BLASTX E-values for target annotation were 1E-20 and 1E-5, respectively. Both protein databases were prepared with plants only.

We measured the efficiency of multi-thread management on Ubuntu 9.10 (karmic) machine with four Intel(R) Core(TM)2 Quad CPU Q6600 at 2.4 GHz, 8GB RAM. The average speed of the miRNA and target identifications on TAIR10 cDNAs with default parameter settings was improved by 30 % and 46 % from single to two and four threads (see Table 4).

**Table 4:** Time usage for each step of miRNA and target identifications on TAIR10 cDNA dataset with varied number of threads running

	1 thread	2 threads	4 threads
**miRNA identification steps**			

Homolog search	2:40:31	1:56:20	1:34:31
Primary miRNA folding	1:39:08	0:55:15	0:34:31
Precursor miRNA folding	0:18:24	0:22:10	0:17:40

**Target identification steps**			

Target scanning	0:22:23	0:20:10	0:19:22
miRA-target folding	0:22:24	0:14:15	0:14:39
Target annotation	0:29:19	0:16:09	0:10:15

* The format of time usage is hours:minutes:seconds

## Conclusions

This paper presents C-mii, a standalone software package for computational identification of plant miRNAs and targets. C-mii has been implemented as all-in-one Java package with following distinguishing features. First, it comes with graphical user interfaces of well-defined pipelines for both miRNA and target identifications, with reliable results. Second, it provides a set of filters allowing users to reduce the number of results corresponding to the recently proposed constraints in plant miRNA and target biogenesis. Third, it extends the standard computational steps of miRNA target identification with an miRNA-target folding module and GO annotation. Fourth, it supplies bird's eye views of the identification results with infographics and grouping information. Fifth, it provides helper functions for database update and auto-recovery to ease system usage and maintenance. Finally, it supports multi-project and multi-thread management to improve computational speed. With these features, C-mii is a very useful software package that can help accelerate the study of plant miRNAs and targets by plant biologists.

## Availability and requirements

1) **Project name: **C-mii: A tool for plant miRNA and target identification

2) **Project home page: **http://www.biotec.or.th/isl/c-mii

3) **Operating system(s): **Windows and Ubuntu Linux 9.10 or higher

4) **Programming language: **Java and Python

5) **Other requirements: **-

6) **License: **GNU GPL

7) **Any restrictions to use by non-academics: **license needed

## Competing interests

The authors declare that they have no competing interests.

## Authors' contributions

SN, WM, SI, and DW together designed software architecture and graphical user interfaces. SN developed Java-based interfaces and modules. WM implemented python scripts for results extractions. DW oversaw the software development. All authors helped test the overall functionalities of the system, and performed benchmarking and validation. SI helped draft the manuscript and DW wrote this manuscript. All co-authors read and approved the final manuscript.

## Supplementary Material

Additional file 1 **FASTA (can be viewed with a text editor) - TAIR10 cDNA sequences**. This file is used as input for benchmarking the miRNA identification pipeline. It is available under the Documentation & Benchmarking menu at http://www.biotec.or.th/isl/c-mii. Click here for file

Additional file 2 **FASTA (can be viewed with a text editor) - TAIR10 miRNA sequences**. This file is used as input for benchmarking the miRNA identification pipeline. Click here for file

Additional file 3 **FASTA (can be viewed with a text editor) - *Arabidopsis *precursor miRNA sequences from miRBase 16**. This file is used as input for benchmarking the miRNA identification pipeline. Click here for file

Additional file 4 **(FASTA) - All plant RNA sequences that are not miRNAs from Rfam 10**. This file is used as input for benchmarking the miRNA identification pipeline. Click here for file

Additional file 5 **(Microsoft Excel) - List of true positives (TP), false positives (FP), and false negatives (FN) of the miRNA identification on TAIR10 cDNA dataset**. Click here for file

Additional file 6 **(Microsoft Excel) - List of false negatives (FN) of the miRNA identification on *Arabidopsis *precursor miRNAs from miRBase 16 dataset**. Click here for file

Additional file 7 **(FASTA) - *Arabidopsis *known miRNA target sequences**. This file is used as input for benchmarking the target identification pipeline. Click here for file
