# Supplementary material for: C-mii: a tool for plant miRNA and target identification
Source: BMC Genomics. 2012 Dec 7;13(Suppl 7):S16. doi: 10.1186/1471-2164-13-S7-S16 (PMC3521235; doi:10.1186/1471-2164-13-S7-S16)
Supplement: Additional file 1 — FASTA (can be viewed with a text editor) - TAIR10 cDNA sequences. This file is used as input for benchmarking the miRNA identification pipeline. It is available under the Documentation & Benchmarking menu at http://www.biotec.or.th/isl/c-mii. [file 1471-2164-13-S7-S16-S1.doc]

### Additional file 1 (FASTA) – TAIR10 cDNA sequences

This file is used as input for benchmarking the miRNA identification.

It is available under the Documentation & Benchmarking menu at <http://www.biotec.or.th/isl/c-mii>.
